# PINK1 Protects against Staurosporine-Induced Apoptosis by Interacting with Beclin1 and Impairing Its Pro-Apoptotic Cleavage

**DOI:** 10.3390/cells11040678

**Published:** 2022-02-15

**Authors:** Francesco Brunelli, Liliana Torosantucci, Vania Gelmetti, Davide Franzone, Anne Grünewald, Rejko Krüger, Giuseppe Arena, Enza Maria Valente

**Affiliations:** 1 Department of Molecular Medicine, University of Pavia, 27100 Pavia, Italy; francesco.brunelli01@universitadipavia.it (F.B.); davide.franzone01@universitadipavia.it (D.F.); 2 Mendel Laboratory, Casa Sollievo della Sofferenza Hospital, IRCCS, 71013 San Giovanni Rotondo, Italy; lilitorosantucci@gmail.com (L.T.); vania.gelmetti@gmail.com (V.G.); 3 Luxembourg Centre for Systems Biomedicine, University of Luxembourg, L-4367 Luxembourg, Luxembourg; anne.gruenewald@uni.lu (A.G.); rejko.krueger@uni.lu (R.K.); 4 Institute of Neurogenetics, University of Lübeck, 23538 Lubeck, Germany; 5 Centre Hospitalier du Luxembourg, Parkinson Research Clinic, L-1210 Luxembourg, Luxembourg; 6 Transversal Translational Medicine, Luxembourg Institute of Health, L-1445 Luxembourg, Luxembourg; 7 Neurogenetics Research Centre, IRCCS Mondino Foundation, 27100 Pavia, Italy

**Keywords:** PINK1, Beclin1, autophagy, apoptosis, neurodegeneration, cancer

## Abstract

*PINK1* is a causative gene for Parkinson’s disease and the corresponding protein has been identified as a master regulator of mitophagy—the autophagic degradation of damaged mitochondria. It interacts with Beclin1 to regulate autophagy and initiate autophagosome formation, even outside the context of mitophagy. Several other pro-survival functions of this protein have been described and indicate that it might play a role in other disorders, such as cancer and proliferative diseases. In this study, we investigated a novel anti-apoptotic function of PINK1. To do so, we used SH-SY5Y neuroblastoma cells, a neuronal model used in Parkinson’s disease and cancer studies, to characterize the pro-survival functions of PINK1 in response to the apoptosis inducer staurosporine. In this setting, we found that staurosporine induces apoptosis but not mitophagy, and we demonstrated that PINK1 protects against staurosporine-induced apoptosis by impairing the pro-apoptotic cleavage of Beclin1. Our data also show that staurosporine-induced apoptosis is preceded by a phase of enhanced autophagy, and that PINK1 in this context regulates the switch from autophagy to apoptosis. PINK1 protein levels progressively decrease after treatment, inducing this switch. The PINK1–Beclin1 interaction is crucial in exerting this function, as mutants that are unable to interact do not show the anti-apoptotic effect. We characterized a new anti-apoptotic function of PINK1 that could provide options for treatment in proliferative or neurodegenerative diseases.

## 1. Introduction

While autophagy and apoptosis are interrelated, they are, at least to some extent, alternative cellular functions that play a crucial role in survival and homeostasis. These processes, essential for post-mitotic cells such as neurons, have been linked to neurodegenerative diseases both from a genetic and a pathophysiological perspective [1,2]. The two most common neurodegenerative diseases, Alzheimer’s disease (AD) and Parkinson’s disease (PD), share common pathophysiological features such as accumulation of misfolded proteins, mitochondrial damage, and synaptic dysfunction, resulting in the death of a subpopulation of neurons—including hippocampal and cortical neurons in AD, nigral dopaminergic neurons in PD. In both diseases, proteins implicated in autophagy have been associated with these pathophysiological features. Approximately 1 in 3 people in Western countries will at some point in life be affected by either AD or PD, and the global burden of these diseases is expected to grow significantly with the increase in life expectancy [3].

PINK1 is a mitochondrial kinase playing a causative role in autosomal recessive PD. Its most well-characterized function is the regulation of mitophagy, the autophagic degradation of damaged mitochondria, but other pro-survival and neuroprotective functions of this protein have been described [4]. It is a positive regulator of cell cycle [5], it participates in maintenance of calcium homeostasis [6,7], and cells depleted of PINK1 show defects in mitochondrial bioenergetics [8]. It is involved in the removal of misfolded proteins through autophagy and the ubiquitin-proteasome system [9], and a recent study demonstrated that enhanced PINK1 activity rescues amyloid pathology in AD [10]. It is the first and most studied ubiquitin kinase [11].

Besides neurodegeneration, an increasing number of experimental results point to a role of PINK1 in proliferative disorders. This is not surprising, as the molecular and cellular pathophysiological mechanisms intervening in these two groups of diseases are, in part, the same, often acting or dysregulated in opposing directions. Some researchers even consider them as “two sides of the same coin” [12,13], and epidemiological data seem to support this inverse association [14,15,16]. Several functions of PINK1 fit well in this picture, including its pro-survival and anti-apoptotic effect, mitophagy, and the regulation of mitochondrial homeostasis [17,18]. Autophagy has been a particularly crucial topic in recent years in cancer research both as a pathological process and as a therapeutic target [19,20]. We previously demonstrated that a direct interaction of PINK1 with Beclin1 is responsible for regulation of autophagy and initiation of autophagosome formation upon mitophagic stimuli [21,22]. In the current study, we show that this PINK1–Beclin1 interaction regulates the balance between autophagy and apoptosis upon treatment with staurosporine (STS), an inducer of apoptosis but not mitophagy. Specifically, our data demonstrate that PINK1 impairs the pro-apoptotic cleavage of Beclin1, favoring an activation of autophagy and preventing the switch towards cell death.

## 2. Materials and Methods

### 2.1. Eukaryotic Expression Vectors and shRNA Constructs

PINK1 and Beclin1 over-expression constructs, both pcDNA3.1-based, have been described previously [21]. Wild-type PINK1 (PINK1-WT), as well as the PD-related PINK1-G309D and PINK1-W437X mutants, were all tagged at the C-terminus with the HA epitope. Beclin1 was tagged at the C-terminus with the Myc epitope. To knockdown PINK1 expression in SH-SY5Y cells (shPINK1), pMISSION validated shRNA bacterial glycerol stocks (NM_032409, TRCN0000199193) were purchased from MERCK (Darmstadt, Germany). Efficiency of PINK1 knockdown in SH-SY5Y cells using this small hairpin RNA was demonstrated previously [23]. The MISSION pLKO.1-puro Non-mammalian shRNA Control Plasmid DNA (SHC002, MERCK), targeting no known mammalian genes, was used as negative control. Lentiviral vectors were amplified in the Stbl3 bacterial strain and used to generate lentiviral particles in 293T packaging cells.

### 2.2. Cell Cultures

SH-SY5Y neuroblastoma cells, as well as the HEK-293T cell line, were maintained in Dulbecco’s modified Eagle’s medium (DMEM; Thermo Fisher Scientific, Waltham, MA, USA), supplemented with 2 mM L-glutamine, 200 U/mL penicillin, 200 mg/mL streptomycin, 1 mM sodium pyruvate and 10% heat inactivated FBS at 37 °C in 95% humidifier air and 5% CO_2_. Parkin-inducible SH-SY5Y cells, kindly provided by Dr. Wolfdieter Springer and Prof. Philippe J Kahle (Laboratory of Functional Neurogenetics, Department for Neurodegenerative Diseases, Hertie Institute for Clinical Brain Research, University of Tübingen, Tübingen, Germany), were cultured in DMEM/HAM F12 1:1 (Thermo Fisher Scientific) containing 10% Tetracycline-free FBS (Takara Bio, San Jose, CA, USA), 7 μg/mL Blasticidin and 300 μg/mL Zeocin (both from Thermo Fisher Scientific). Parkin expression was induced by adding 1 μg/mL Doxicycline (MERCK) for 48 h [24].

### 2.3. Transfections and Transductions

pcDNA3.1-based constructs were expressed in SH-SY5Y cells upon transfection with Lipofectamine 2000 reagent (Thermo Fisher Scientific). Stable transfectants expressing either PINK1-WT or the PD-related PINK1-G309D and PINK1-W437X mutants, as well as Beclin1, were selected as previously described [21]. SH-SY5Y cells stably expressing GFP-LC3 were also described previously [21]. shRNA delivery into SH-SY5Y cells was achieved by means of lentiviral vector-mediated transfer. Lentiviral constructs (control-shRNA or PINK1-shRNA) as well as 2nd generation packaging plasmids (psPAX2, pMD2G), were transfected in HEK293T cells by calcium phosphate precipitation in presence of 25 µm chloroquine. HEK293T cell culture medium, containing the respective lentiviral particles, was harvested 48 h post-transfection, passed through 0.45 µm filters and used to transduce target cells overnight, in the presence of 8 µg/mL Polybrene (MERCK). Stable transductants were obtained by adding 2 μg/mL Puromycin (MERCK) up to 10 days.

### 2.4. Treatments

SH-SY5Y cells were exposed to 1 µM Staurosporine (MERCK), 20 µM CCCP (MERCK), 1 µM Geldanamycin (MERCK), 50 μg/mL Etoposide (MERCK) or vehicle (DMSO) at the indicated times. z-VAD-fmk (MERCK) was used at the final concentration of 50 µM for 3 h. MG132 (MERCK) was used at the final concentration of 10 µM for 12 h.

### 2.5. Antibodies

The following antibodies were used for Western blotting and immunofluorescence assays: mouse anti-HA (MERCK), rabbit anti-HA (MERCK), mouse anti-Myc (Santa-Cruz Biotechnology, Santa Cruz, CA, USA), rabbit anti-PINK1 (Novus Biologicals, Englewood, CO, USA), mouse anti-TIM23 (BD Biosciences, Franklin Lakes, NY, USA), mouse anti-TOM20 (BD Biosciences), mouse anti-Parkin (Santa-Cruz Biotechnology), rabbit anti-GAPDH (Cell Signaling Technologies, Danvers, MA, USA), rabbit anti-LC3B (MERCK) and rabbit anti-cleaved PARP (Cell Signaling Technologies). HRP-conjugated secondary antibodies were as follows: anti-Mouse and anti-Rabbit (both from GE Healthcare, Little Chalfont, UK). Secondary antibodies used in immunofluorescence experiments were conjugated with either Alexa Fluor 488, Alexa Fluor 555 or Alexa Fluor 405 (Thermo Fisher Scientific).

### 2.6. Western Blotting Analysis

Cells were lysed in RIPA buffer (Cell Signaling Technologies) containing protease and phosphatase inhibitors and protein extracts were quantified by using the bicinchoninic acid (BCA) assay (Thermo Fisher Scientific). Lysates (50 μg) were subjected to SDS-PAGE, probed with the primary and secondary antibodies listed above, and detected by using ECL-Plus Western Blotting Detection System (GE Healthcare). All experiments were normalized by GAPDH expression. Image contrast and brightness, as well as densitometry measurements, were performed in Adobe Photoshop CS2 (Adobe Systems Incorporated, San Jose, CA, USA). In all panels, values are plotted as fold-change relative to control, which has been set to a value of 1.

### 2.7. Immunofluorescence and Confocal Microscopy

Immunofluorescence analysis was performed as described previously [21]. Images were acquired by using the confocal microscope PCM Eclipse TE300 or the C2 Confocal Microscopy System (both from Nikon Instruments, Tokyo, Japan). Merged images were obtained with EZ2000 or NIS Element software. Quantification of colocalization, expressed in terms of overlap coefficient (R), was calculated on several randomly selected cells from different slides by using the WCIF ImageJ software.

### 2.8. RNA Extraction and RT-qPCR

RNA was extracted using the RNeasy Mini Kit (Qiagen, Hilden, Germany), including on-column DNase I digestion, according to the manufacturer’s instructions. An amount of 1 µg of RNA was retro-transcribed using the High-Capacity cDNA Reverse Transcription Kit (Thermo Fisher Scientific). Quantitative PCR was performed using iQ SYBR Green Supermix (Bio-Rad Laboratories, Hercules, CA, USA) on the LightCycler 480 instrument. PINK1 primer sequences have been previously published [22]. *PINK1* relative gene expression was normalized to *β-actin* and calculated using the 2 ^−ΔΔCT^ method.

### 2.9. Statistical Analysis

Densitometric results of immunoblotting, as well as confocal microscopy measurements were represented as histograms; values were obtained from at least three independent experiments and expressed as means ± S.E.M. Statistical analysis was carried out by using unpaired two-tailed Student’s *t*-test, with *p*-values < 0.05 considered as significant.

## 3. Results

### 3.1. Autophagy Induced by STS Treatment Precedes Apoptotic Cell Death

STS has long been used as an inducer of apoptosis in many different cell types including dopaminergic neuronal cell lines [25,26,27]. However, several studies have reported a parallel activation of the autophagic response following STS treatment, showing conflicting results regarding the influence of autophagy on cell death [27,28].

**Figure 1:**
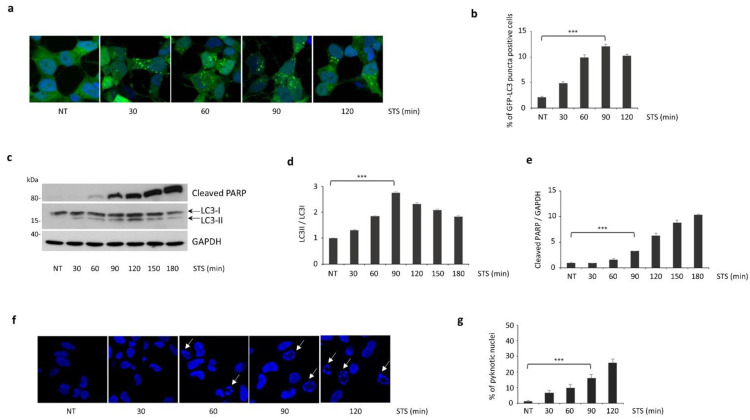
STS treatment induces autophagosome enhancement followed by a progressive activation of apoptosis in SH-SY5Y cells. (**a**) Representative pictures of SH-SY5Y cells stably expressing GFP–LC3 treated with either STS (at the indicated time points) or DMSO (NT); (**b**) the count of cells showing GFP–LC3 autophagic vacuoles (puncta) showed maximum autophagy activation at 90 min; (**c**) in immunoblots, autophagy induction was monitored analyzing the conversion of endogenous LC3 from the cytoplasmic (LC3-I, 18 kDa) to the membrane-bound form (LC3-II, 16 kDa), while apoptosis was monitored by cleaved PARP; (**d**) densitometric analysis of LC3-II/LC3-I ratio showed a progressive increment until a three-fold increase at 90 min (2.74 ± 0.06, *p* = 0.0008); (**e**) densitometric analysis of cleaved PARP showed a significant increase since 90 min from STS exposure (3.31 ± 0.003, *p* = 0.0001). GAPDH protein levels were used for normalization; (**f**) Western blot data were confirmed by confocal analysis of apoptotic cells, identified by irreversible chromatin condensation within pyknotic nuclei (white arrows). (**g**) The count of pyknotic nuclei showed a significant increase over time after STS treatment. Experiments performed in triplicate; a minimum of 200 cells per experiment were analyzed; mean ± S.E.M.). *** *p* ≤ 0.001.

To assess the induction of autophagy and apoptosis in our cellular model, SH-SY5Y cells were treated with either 1 μM STS or vehicle (DMSO) and markers of both processes were analyzed at different time points up to 3 h. As expected, STS was able to activate the autophagy pathway, as shown by the progressive increase in LC3-positive vacuoles in SH-SY5Y cells over-expressing GFP-LC3 (Figure 1a,b). A similar result was observed in immunoblotting experiments showing a significant accumulation of the autophagosomal marker LC3-II as early as 30 min after STS exposure. Of note, LC3-II reached the maximum level at about 90 min and subsequently decreased for up to 3 h (Figure 1c,d). In this experimental setting, STS also increased apoptosis, as demonstrated by the gradual increment of the 89 kDa caspase-dependent cleaved fragment of PARP and the increased number of pyknotic nuclei, but only starting from 90 min post-treatment (Figure 1c,e–g). Altogether, these observations indicate that, upon STS treatment, autophagy induction precedes the activation of apoptosis in SH-SY5Y cells.

### 3.2. PINK1 Protein Levels Decrease during STS Treatment

**Figure 2:**
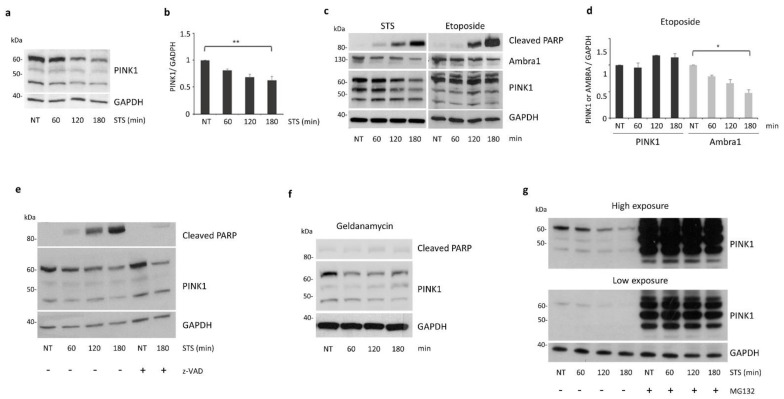
PINK1 protein levels decrease during STS treatment. (**a**) SH-SY5Y cells transiently overexpressing PINK1-HA were treated either with STS (for 1, 2 and 3 h) or DMSO for 3 h (NT) and then processed for Western blotting by using an anti-PINK1 antibody (Novus). Equal loading was verified by GAPDH protein levels; (**b**) densitometric analysis showing a progressive decrease in full-length (FL) PINK1 levels during treatment; (**c**) SH-SY5Y cells co-expressing Ambra-Myc and PINK1-HA were treated with STS or etoposide at the indicated times and analyzed by immunoblotting. A similar increase in cleaved PARP levels was observed after STS or etoposide exposure; however, even if Ambra protein levels decreased in both experimental conditions, FL-PINK1 levels decreased significantly only upon STS treatment; (**d**) densitometric analysis of PINK1 and Ambra proteins in SH-SY5Y cells treated with etoposide; (**e**) SH-SY5Y cells transiently expressing PINK1-HA were treated with STS or with both the caspase inhibitor z-VAD-fmk and STS for 3 h. Immunoblot analysis with the anti-PINK1 antibody showed a comparable decrease in FL-PINK1 levels for 3 h of STS or z-VAD-fmk/STS treatment, excluding an involvement of caspase in PINK1 degradation; (**f**) lysates from SH-SY5Y cells, treated with Geldanamycin (Hsp90 inhibitor) at the indicated times, were analyzed by immunoblotting with the anti-PINK1 antibody to monitor FL-PINK1 protein levels; (**g**) SH-SY5Y cells transiently expressing PINK1-HA were treated with STS ± MG132 (proteasome inhibitor). Immunoblot analysis with the anti-PINK1 antibody showed a strong stabilization of HA-PINK1 after MG132 treatment. * *p* ≤ 0.05, ** *p* ≤ 0.01.

**Figure A1:**
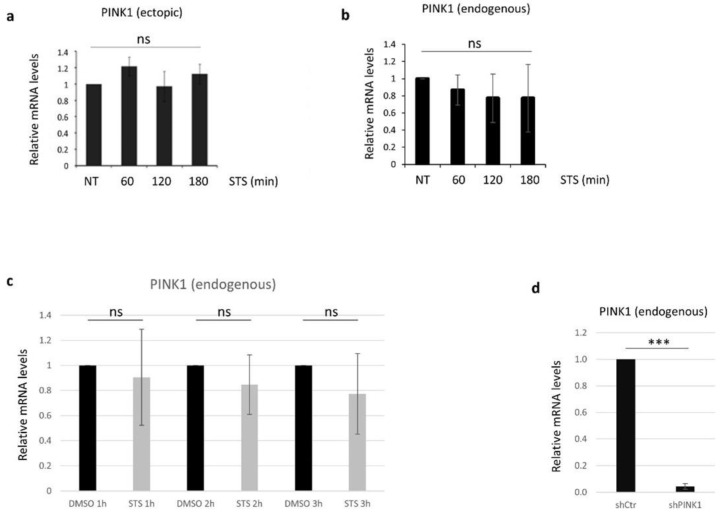
PINK1 gene transcription does not change significantly after STS exposure, while it is significantly reduced after shPINK1. (**a**) Analysis of PINK1 mRNA levels was performed by RT-qPCR in PINK1-overexpressing SH-SY5Y cells. We observed no significant differences in PINK1 transcript at the indicated times; (**b**) analysis of endogenous PINK1 mRNA levels was performed by RT-qPCR. We observed no significant differences in PINK1 expression after STS treatment for the indicated times. “NT” refers to cells treated for DMSO for 3 h; (**c**) the analysis of endogenous PINK1 mRNA by RT-qPCR did not show significant differences in a point-by-point comparison between SH-SY5Y cells treated either with DMSO or with STS for 1, 2, and 3 h; (**d**) PINK1 downregulation by shRNA (shPINK1) induces a significant reduction in endogenous PINK1 mRNA in SH-SY5Y cells. *** *p* ≤ 0.001. ns = not significant.

It has been previously demonstrated that PINK1 prevents cell death induced by STS treatment [25,29,30]. Besides this, PINK1 is also able to promote basal and starvation-induced autophagy [21]. As several pro-autophagic proteins are down-regulated during apoptosis, which leads to autophagy inhibition in favor of the apoptotic process [31], we asked whether PINK1 could be involved in this process. To this end, we first monitored PINK1 protein levels in SH-SY5Y cells treated with STS. Cells transiently overexpressing PINK1 displayed a gradual and significant decrease in full-length (FL) PINK1 protein during STS treatment (Figure 2a,b). In this setting, PINK1 mRNA levels remained unchanged (Appendix, Figure A1a). Similarly, mRNA levels of endogenous PINK1 did not vary significantly in SH-SY5Y cells treated with STS up to 3 h, thus indicating a post-transcriptional regulation of PINK1 upon STS treatment (Appendix, Figure A1b,c).

Since another key regulator of autophagy and apoptosis, namely the Ambra1 protein, is cleaved and degraded upon STS treatment [32], we hypothesized that PINK1 could be regulated in a similar manner. However, despite Ambra1 protein levels decreased both in STS- and etoposide-treated cells, no significant differences were observed upon etoposide exposure for FL-PINK1 (Figure 2c,d). In addition, incubation of SH-SY5Y cells with the pan caspase inhibitor z-VAD-fmk failed to prevent PINK1 down-regulation induced by STS treatment, thus indicating a caspase-independent mechanism responsible for PINK1 degradation (Figure 2e).

It has been previously reported that PINK1 protein stability depends on the ATPase activity of the Cdc37/Hsp90 molecular chaperone. Thus, inhibition of this machinery by molecules able to interfere with the ATP/ADP binding site of Hsp90, such as Geldanamycin, could destabilize its client proteins, including PINK1 [33,34]. Notably, STS is also able to inhibit the activity of protein kinases through competition for their ATP binding site [35]. According to this notion, we found a similar PINK1 degradation pattern upon Geldanamycin and STS treatment (Figure 2a,f), indicative of a reduced stability of FL-PINK1 protein following STS exposure. As the 54 kDa form of PINK1 undergoes constitutive degradation by the proteasome in the cytoplasm [34], we hypothesized that the same pathway could be responsible for the degradation of the FL protein. In support of this hypothesis, we demonstrated that, in presence of the proteasome inhibitor MG132, PINK1 protein levels were not affected by STS treatment (Figure 2g).

### 3.3. PINK1 Degradation Controls the Switch between STS-Induced Autophagy and Apoptosis

Based on our previous results, we hypothesized that the increased apoptosis observed upon STS treatment could be the consequence of reduced autophagy activation due to a decline in PINK1 protein levels.

**Figure 3:**
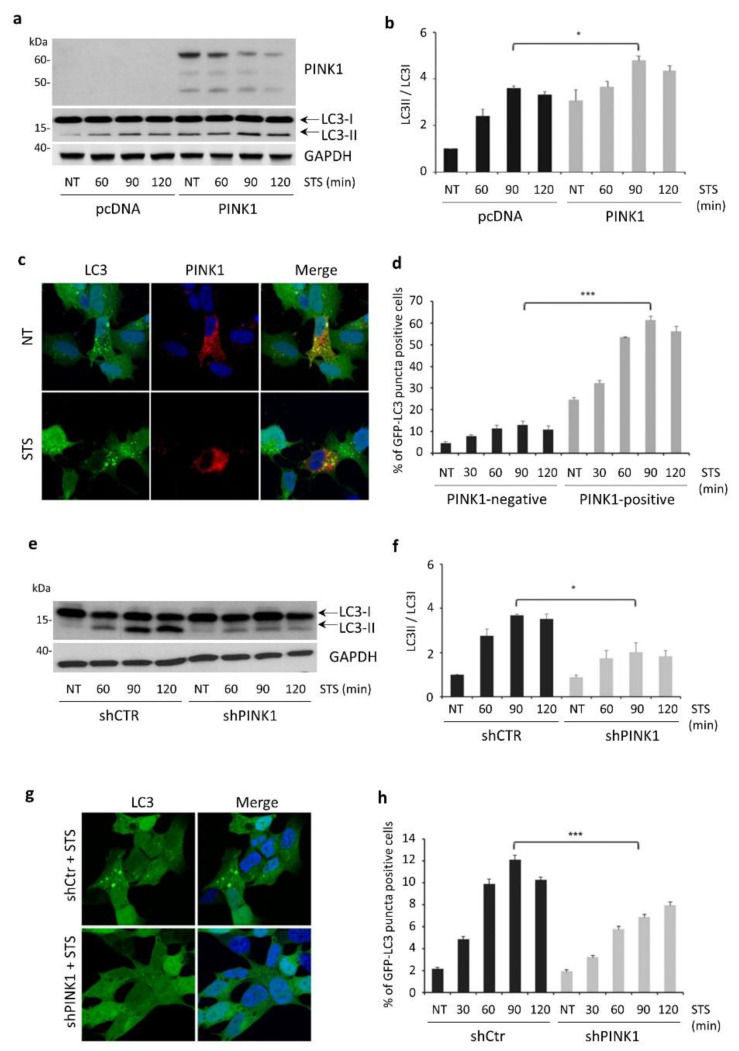
PINK1 expression regulates STS-induced autophagy. (**a**) Western blotting analysis of the autophagic marker LC3 in SH-SY5Y cells transiently transfected with PINK1-FL or vector alone (pcDNA) and then treated either with STS (for 1, 2 and 3 h) or with DMSO for 3 h (NT). PINK1 expression was verified by immunoblot analysis with an anti-PINK1 antibody. Equal loading was verified by GAPDH protein levels; (**b**) the LC3-II/LC3-I ratio in cells overexpressing PINK1 was significantly higher than in cells transfected with the empty vector (pcDNA) after 90 min of STS exposure (3.33 ± 0.13 versus 4.37 ± 0.22, *p* = 0.0158); (**c**) these data were confirmed by the higher number of cells showing GFP-LC3 autophagic vacuoles (in green) in presence of PINK1 over-expression (in red), analyzed by confocal microscopy after 90 min of STS treatment; (**d**) GFP-LC3 autophagic vacuoles were more abundant in cells overexpressing PINK1 (PINK1-positive) compared to untransfected cells (PINK1-negative). The effect, already observed in basal conditions, further increased upon treatment with STS for 90 min (10.8 ± 1.92 versus 56.2 ± 2.12, *p* = 0.0001); (**e**) Western blotting analysis of the autophagic marker LC3 in SH-SY5Y cells transduced with PINK1-shRNA (shPINK1) or control-shRNA (shCtr) and then treated either with STS (for 1, 2 and 3 h) or with DMSO for 3 h (NT). Equal loading was verified by GAPDH protein levels; (**f**) densitometric analysis of (**e**) showing a significant reduction in the LC3-II/LC3-I ratio in cells transduced with shPINK1 compared to shCtr after 90 min of STS treatment (3.52 ± 0.22 versus 1.83 ± 0.27, *p* = 0.0259); (**g**) these data were confirmed by the number of positive cells for GFP-LC3 autophagic vacuoles analyzed by confocal microscopy after 90 min of STS treatment; (**h**) At this time point, the count of cells positive for GFP-LC3 autophagic vacuoles was significantly lower in cells transduced with shPINK1 compared to shCtr (12.1 ± 0.24 versus 6.2 ± 0.28, *p* = 0.0001). * *p* ≤ 0.05, *** *p* ≤ 0.001.

To test this hypothesis, we first analyzed autophagy activation in SH-SY5Y cells transiently transfected with PINK1-FL. During STS exposure, PINK1-FL overexpression significantly induced autophagy compared to cells transfected with vector alone, as indicated by the increased LC3-II/LC3-I ratio in immunoblotting experiments (Figure 3a,b). Accordingly, the percentage of cells showing LC3-positive vacuoles strongly increased in PINK1-FL overexpressing cells (Figure 3c,d). In contrast, PINK1 down-regulation by using a previously validated shRNA (Appendix, Figure A1d) resulted in a significant decrease in STS-induced autophagy compared to control cells (Figure 3e–h).

**Figure 4:**
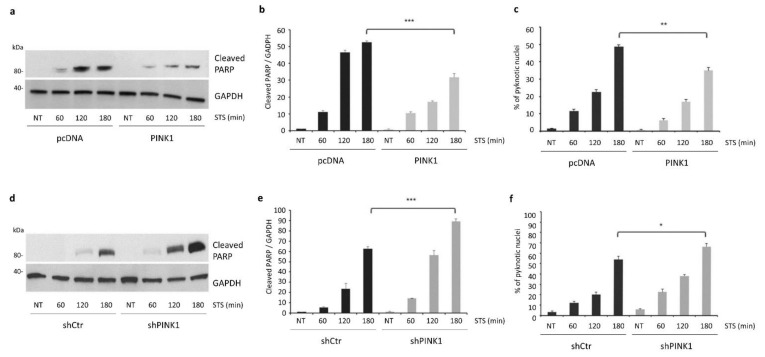
PINK1 protects from STS-induced cell death. (**a**) Immunoblot analysis of PARP cleavage in SH-SY5Y cells overexpressing PINK1 or vector alone (pcDNA) and then treated either with STS (for 1, 2 and 3 h) or with DMSO for 3 h (NT). For PINK1 expression in the same experimental conditions, see Figure 3a. Equal loading was verified by GAPDH protein levels; (**b**) densitometric analysis of (**a**) showing decreased levels of cleaved PARP in STS-treated PINK1 over-expressing cells compared to cells transfected with the empty vector (pcDNA) (31.77 ± 2.1 versus 52.54 ± 0.85, *p* = 0.001); (**c**) confocal analysis of apoptotic nuclei confirmed data obtained in immunoblotting experiments (48.66 ± 1.2 versus 35 ± 1.7, *p* = 0.0028); (**d**) immunoblot analysis of PARP cleavage in SH-SY5Y cells transduced with PINK1-shRNA (shPINK1) or control-shRNA (shCtr) and then treated either with STS (for 1, 2 and 3 h) or with DMSO for 3 h (NT). Equal loading was verified by GAPDH protein levels; (**e**) densitometric analysis of (**d**) showing a significant increase in cleaved PARP levels in shPINK1 cells compared to shCtr after 180 min of STS exposure (62.6 ± 2.28 versus 89.1 ± 2.5, *p* = 0.001); (**f**) a similar increase was observed through confocal microscopy analysis of apoptotic nuclei (54 ± 3.2 versus 66.3 ± 3.2, *p* = 0.00527). * *p* ≤ 0.05, ** *p* ≤ 0.01, *** *p* ≤ 0.001.

In line with our previous results, markers of apoptosis were significantly induced upon STS exposure, in particular at 2–3 h post-treatment, which corresponds to the time points characterized by decreased PINK1 stability and reduced autophagy activation. In fact, transient expression of PINK1-FL in SH-SY5Y cells significantly impaired apoptotic cell death induced by STS treatment, as revealed by decreased levels of cleaved PARP and a reduced percentage of cells with pyknotic nuclei (Figure 4a–c). Conversely, PINK1 knockdown further accelerated STS-induced apoptosis, which became significant as soon as 3 h after STS exposure (Figure 4d–f).

### 3.4. Absence of Mitophagy Induction in Response to STS Treatment

**Figure 5:**
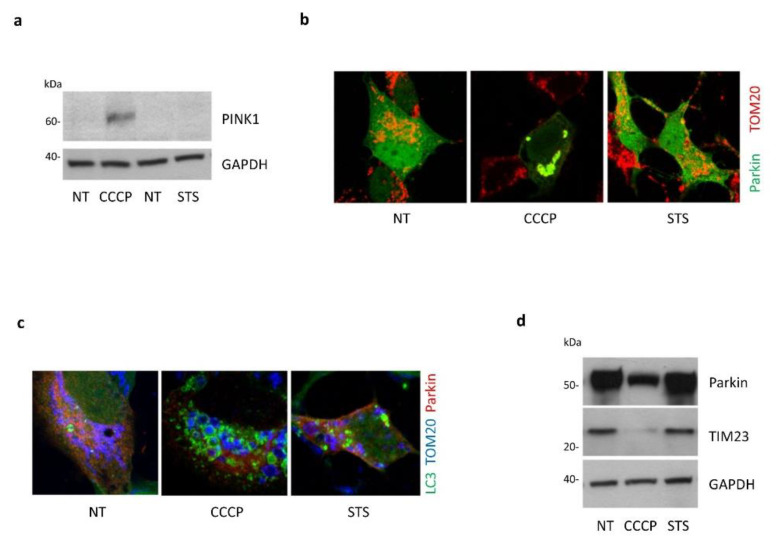
STS treatment does not induce PINK1 mediated mitophagy in SH-SY5Y cells. (**a**) SH-SY5Y cells were treated either with CCCP (360 min), STS (120 min) or vehicle (NT). Accumulation of endogenous PINK1 was only detected by Western blot after CCCP treatment, but not after STS treatment. Equal loading was verified by GAPDH protein levels; (**b**) Parkin-inducible SH-SY5Y cells were treated either with CCCP (360 min) or STS (120 min) and Parkin recruitment at mitochondria was assessed by confocal microscopy analysis. Parkin was immunostained with HA antibody (green); TOM-20 antibody was used to label the outer mitochondrial membrane (red). Parkin colocalized with TOM-20 only upon CCCP, whereas no colocalization was observed upon STS treatment; (**c**) colocalization of autophagosomes (LC3) and mitochondria (TOM-20). SH-SY5Y cells stably expressing GFP–LC3 were transiently transfected with Parkin-HA, treated with 360 min CCCP or 180 min STS and subjected to confocal microscopy analysis. Parkin and TOM-20 were immunostained in red and blue, respectively. Upon STS treatment, the number of cells with mitochondria engulfed into autophagosomes was significantly lower than in CCCP-treated cells; (**d**) mitophagy was also evaluated by assessing the expression of the mitochondrial marker TIM-23 by Western blotting, which significantly decreased only in Parkin-inducible SH-SY5Y cells treated with CCCP for 360 min. We observed no significant differences in TIM-23 levels upon STS for 360 min. Equal loading was verified by GAPDH protein levels.

It has been previously reported that STS can simultaneously induce autophagy and mitophagy in dopaminergic neuronal cells [27]. We thus assessed whether PINK1 could exert its protective role against STS-induced cell death by activating mitophagy. To this end, we analyzed different mitophagic markers in SH-SY5Y cells treated with STS or with the mitochondrial uncoupler CCCP, the latter as a positive control of mitophagy. Immunoblotting analysis revealed an accumulation of endogenous PINK1 protein upon CCCP exposure, as expected, but not following STS treatment (Figure 5a). Accordingly, confocal microscopy analysis in CCCP-treated Parkin-inducible SH-SY5Y cells [24] showed a strong co-localization between Parkin and the outer mitochondrial membrane marker TOM20, indicative of Parkin recruitment to mitochondria (an early step in mitophagy execution); on the contrary, Parkin displayed a typical cytosolic distribution in cells subjected to STS treatment, suggesting absence of mitophagy (Figure 5b). In line with this, co-localization between mitochondria and the autophagic marker LC3, indicative of mitochondrial incorporation into autophagosomes, was only observed after CCCP but not upon STS treatment (Figure 5c). Finally, we assessed mitochondrial mass by immunoblotting analysis. As shown in Figure 5d, TIM23 levels were clearly reduced as soon as 6 h after CCCP exposure in Parkin-inducible SH-SY5Y cells, whereas they remained unchanged following STS treatment.

### 3.5. STS-Induced Autophagy Is Regulated by PINK1–Beclin1 Interaction

**Figure 6:**
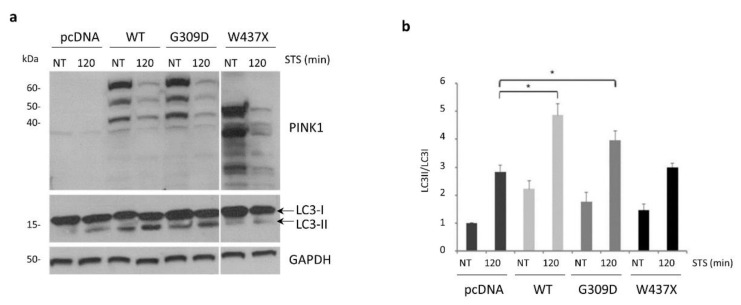
PINK1–Beclin1 interaction regulates STS-induced autophagy. (**a**) PINK1-silenced (shPINK1) SH-SY5Y cells were transiently transfected with empty vector (pcDNA), PINK1 wild-type (WT), PINK1 G309D or PINK1 W437X, and then treated either with STS or with DMSO for 2 h (NT). Autophagy activation was measured by analyzing the LC3-II/LC3-I ratio. PINK1 expression was verified by immunoblot analysis with an anti-PINK1 antibody. Equal loading was verified by GAPDH protein levels. (**b**) Densitometric analysis of (**a**). Both WT-PINK1 and the G309D mutant significantly rescued autophagy impairment, but not the W437X-PINK1 mutant that is unable to interact with Beclin1 (2.84 ± 0.24 versus 3 ± 0.14) [21]. Experiments performed in triplicate; mean ± S.E.M. * *p* ≤ 0.05.

In light of our previous findings showing activation of basal- and starvation-induced autophagy by PINK1 through its interaction with Beclin1 [21], we hypothesized that the same pathway could regulate autophagy induced by STS treatment. To test this hypothesis, we performed complementation experiments in SH-SY5Y cells depleted for PINK1 (shPINK1), by over-expressing PINK1 wild-type protein (WT) or two PD-associated pathogenic mutants characterized by a different capacity to bind Beclin1 [21]. Strikingly, STS-induced autophagy was not affected by over-expressing the PINK1-G309D mutant that maintains the ability to interact with Beclin1 [21], as demonstrated by a similar LC3-II/LC3-I ratio in shPINK1 cells complemented with WT- or G309D-PINK1. Conversely, over-expression of the PINK1-W437X mutant defective for the binding to Beclin1 [21] significantly impaired autophagy induced by STS treatment (Figure 6a,b).

### 3.6. PINK1 Impairs the Pro-Apoptotic Cleavage of Beclin1 upon STS Treatment

**Figure 7:**
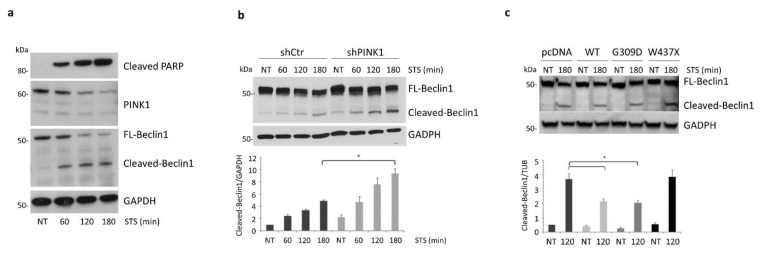
PINK1 protects against Beclin1 pro-apoptotic cleavage in response to STS treatment. (**a**) SH-SY5Y overexpressing PINK1-HA and Beclin1-Myc were treated either with STS (for 1, 2 and 3 h) or with DMSO for 3 h (NT). The progressive formation of the pro-apoptotic fragment of Beclin1 (cleaved-Beclin1) in response to STS treatment was visualized by immunoblotting with the anti-Myc antibody. PINK1 expression was verified by immunoblot analysis with an anti-PINK1 antibody. Equal loading was verified by GAPDH protein levels; (**b**) SH-SY5Y cells stably overexpressing Beclin1-Myc were transduced with PINK1-shRNA (shPINK1) or control-shRNA (shCtr) and then treated either with STS (for 1, 2 and 3 h) or with DMSO for 3 h (NT). Immunoblotting analysis of the caspase-cleaved fragment of Beclin1 (detected by Myc immunostaining) revealed a significant increase in the pro-apoptotic fragment at 180 min of STS in shPINK1 compared to shCtr cells (4.87 ± 0.19 versus 9.34 ± 0.76, *p* = 0.0047). Equal loading was verified by GAPDH protein levels; (**c**) PINK1-silenced (shPINK1) SH-SY5Y cells were transiently transfected with empty vector (pcDNA), PINK1 wild type (WT), PINK1 G309D or PINK1 W437X (together with Beclin1-Myc), and then treated either with STS or with DMSO for 2 h (NT). Beclin1 cleavage was assessed by immunoblotting analysis with the anti-Myc antibody. Equal loading was verified by GAPDH protein levels. Complementation with both WT-PINK1 and the G309D mutant significantly reduced Beclin1 cleavage induced by STS exposure, whereas the PINK1 W437X mutant exhibited levels of cleaved Beclin1 similar to those observed in pcDNA-transfected cells (3.92 ± 0.96 versus 4.03 ± 1.04). * *p* ≤ 0.05.

Several groups highlighted the role of Beclin1 in the crosstalk between apoptosis and autophagy following exposure to apoptotic stressors. For instance, in STS-treated cells, Beclin1 is known to be cleaved by caspase 3, generating a C-terminal fragment that inhibits autophagy and induces cell death [36,37]. Based on these notions and given the key role of PINK1–Beclin1 interaction in autophagy activation, we asked whether the protective function of PINK1 against STS-induced apoptosis could be mediated by the impairment of Beclin1 cleavage. To this aim, we first assessed the production of cleaved-Beclin1 in STS-treated SH-SY5Y cells over-expressing HA-tagged PINK1 and Myc-tagged Beclin1. Strikingly, Beclin1 cleavage was observed as soon as 1 h after STS exposure and progressively increased with time, concurrently with PINK1 protein levels decrease and apoptosis induction (Figure 7a). Importantly, Beclin1 cleavage significantly increased in PINK1-depleted SH-SY5Y cells over-expressing Myc-tagged Beclin1 and subjected to STS treatment (Figure 7b). Finally, to evaluate whether the direct interaction between PINK1 and Beclin1 could be responsible for the inhibition of Beclin1 cleavage during STS treatment, we performed complementation experiments in PINK1-depleted SH-SY5Y cells over-expressing PINK1-WT or the pathogenic mutants PINK1-G309D and PINK1-W437X. In line with our hypothesis, over-expression of both PINK1-WT and PINK1-G309D but not of the mutant construct PINK1-W437X, significantly decreased the amount of cleaved-Beclin1 (Figure 7c).

## 4. Discussion

Since its discovery as a causative gene in PD [38], *PINK1* has been studied in the context of several neurodegenerative diseases [10,39]. The detailed cellular mechanisms by which its deficiency causes neurodegeneration, however, remain elusive. At the same time, several studies have shown that this protein may play a relevant role in oncogenesis, progression and aggressivity of neoplastic diseases [17,18,40,41]. The first and best-characterized cellular function of PINK1 is the regulation of mitophagy, the autophagic degradation of damaged mitochondria. In this setting, PINK1 accumulates on the surface of depolarized mitochondria, where it phosphorylates ubiquitin, activates the E3 ligase Parkin, and recruits autophagy receptors, eventually causing the disposal of malfunctioning organelles [42]. Various studies based on iPSC-derived and animal models, however, questioned the relevance of PINK1-dependent mitophagy in neurons [43,44,45].

We previously showed that PINK1 directly interacts with Beclin1 to promote basal and starvation-induced autophagy, besides mitophagy [21]. This interaction, crucial for the timely accomplishment of the autophagic process, takes place at the ER membranes associated with mitochondria, the site of origin of autophagosomes [22].

In this study we used SH-SY5Y neuroblastoma cells, a model for cancer and PD [46], to demonstrate that PINK1 impairs the pro-apoptotic cleavage of Beclin1, tipping the balance towards autophagy after STS treatment. This observation not only clarifies and strengthens the connection between autophagy and neurodegeneration, but also provides an input to further characterize the role of PINK1 in proliferative diseases and cancer. Our results are consistent with a number of studies supporting a neuroprotective and anti-apoptotic role of PINK1 [4,47,48]. A recent article described the interaction of the co-chaperone STUB1/CHIP with PINK1, which leads to PINK1 ubiquitination and degradation by the proteasome upon STS treatment [49]. This is not in contrast with our findings, but rather complementary, as the interaction with STUB1/CHIP apparently occurs upstream of PINK1, while the interaction with Beclin1 is downstream. In other words, the cited study failed to explain how PINK1 degradation results in increased apoptosis, while ours missed the specific protein responsible for PINK1 degradation.

We also provided a clarification of the cellular processes that follow STS treatment, a subject on which previous research yielded conflicting results [27]. We showed that autophagy and apoptosis are not activated simultaneously, but this happens in a strict chronological sequence. Our experimental setting demonstrated the existence of two alternative phenotypical phases following STS treatment, a pro-autophagic and a pro-apoptotic one, characterized by distinct protein levels and immunochemical markers, in line with earlier hypotheses [31]. PINK1 levels steadily decreased throughout this process, in accordance with the results of previous studies [49]; the described mechanism of PINK1 degradation by the proteasome due to STUB1/CHIP ubiquitination fits well in our experimental findings. In addition, we proved that PINK1 degradation is responsible for the switch from the pro-autophagic to the pro-apoptotic phenotype.

We further determined that the mitophagic process is not activated following STS treatment, as mitophagy markers were not increased and mitochondrial mass was not reduced in our conduct experiments. This clearly advocates for a relevant function of PINK1 beyond mitophagy. Finally, we provided abundant data to support the hypothesis that the PINK1–Beclin1 interaction is crucial in STS-induced autophagy, including complementation experiments in which wild-type PINK1 and two well-characterized pathogenic mutants were expressed in PINK1-depleted cells. Strikingly, the kinase-deficient PINK1-G309D, which is still able to interact with Beclin1 [21], was able to rescue the phenotype as the wild-type protein, while the truncated PINK1-W437X was not. This fitted our current hypothesis and confirmed our past observation that PINK1 directly interacts, and does not phosphorylate, Beclin1 [21,22]. It was previously shown that STUB1/CHIP is able to cause degradation of the PINK1 mutants G309D and L347P [49]. However, the fact that these mutants are degraded in the same manner as the wild-type protein does not imply an effect (or a defect) in activating downstream pathways. Therefore, this is not in contrast with our data.

The use of cancer cell lines recombinantly overexpressing PINK1 has been called into doubt since some results obtained in immortalized cells and fibroblasts could not be reproduced in iPSC-derived neurons [43,50]. This is to some degree an inevitable arrangement to face the difficulties in visualizing endogenous PINK1, and we avoided exogenous expression when feasible. Moreover, it is of note that experiments which showed contrasting results in neurons generally involved PINK1-induced mitophagy, a process whose activation we excluded in the present setting. Finally, and most importantly, any molecular function of PINK1 could be relevant in other CNS cellular populations or in diseases other than neurodegeneration.

Efforts to target PINK1 with candidate therapeutics for neurodegenerative diseases have so far focused on its kinase activity [51]. We believe that its interaction with Beclin1 could provide an alternative for pharmacological treatment. Our present study identified and characterized a novel anti-apoptotic function of this versatile protein, which is potentially amenable to treatment in cancer and neurodegeneration.

## Data Availability

Data presented in this study are available on request from the corresponding authors.
